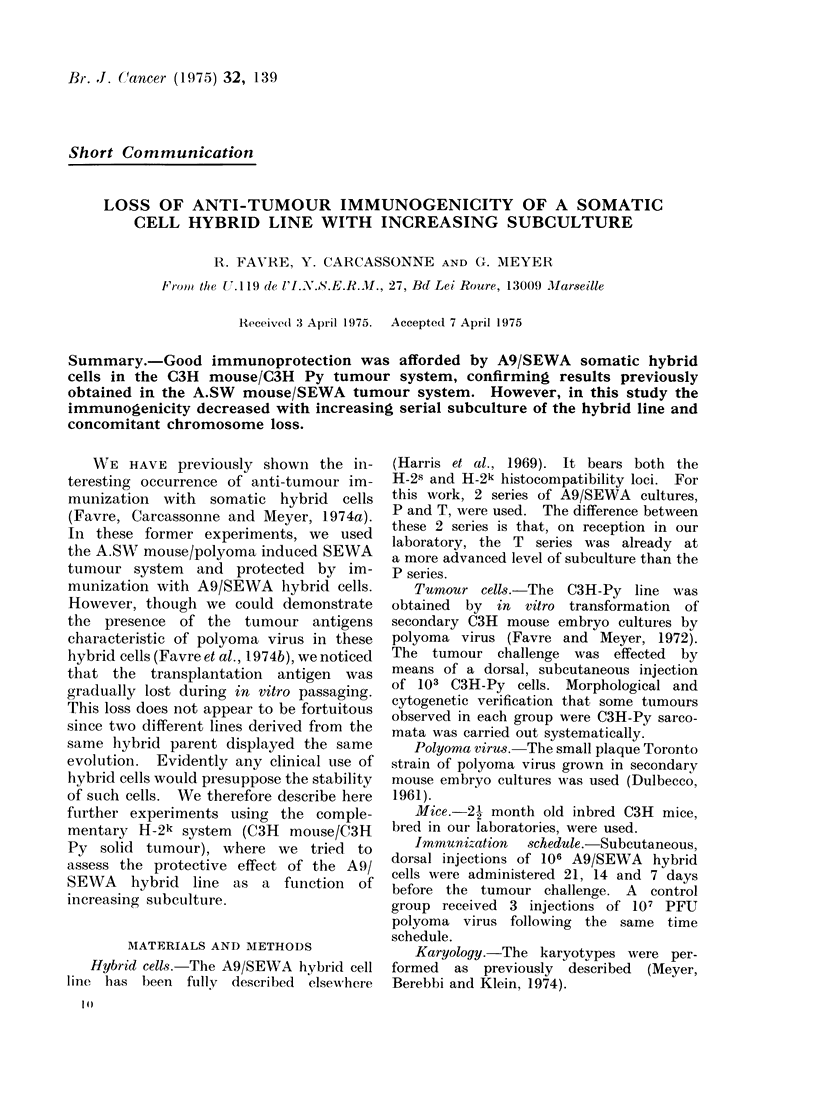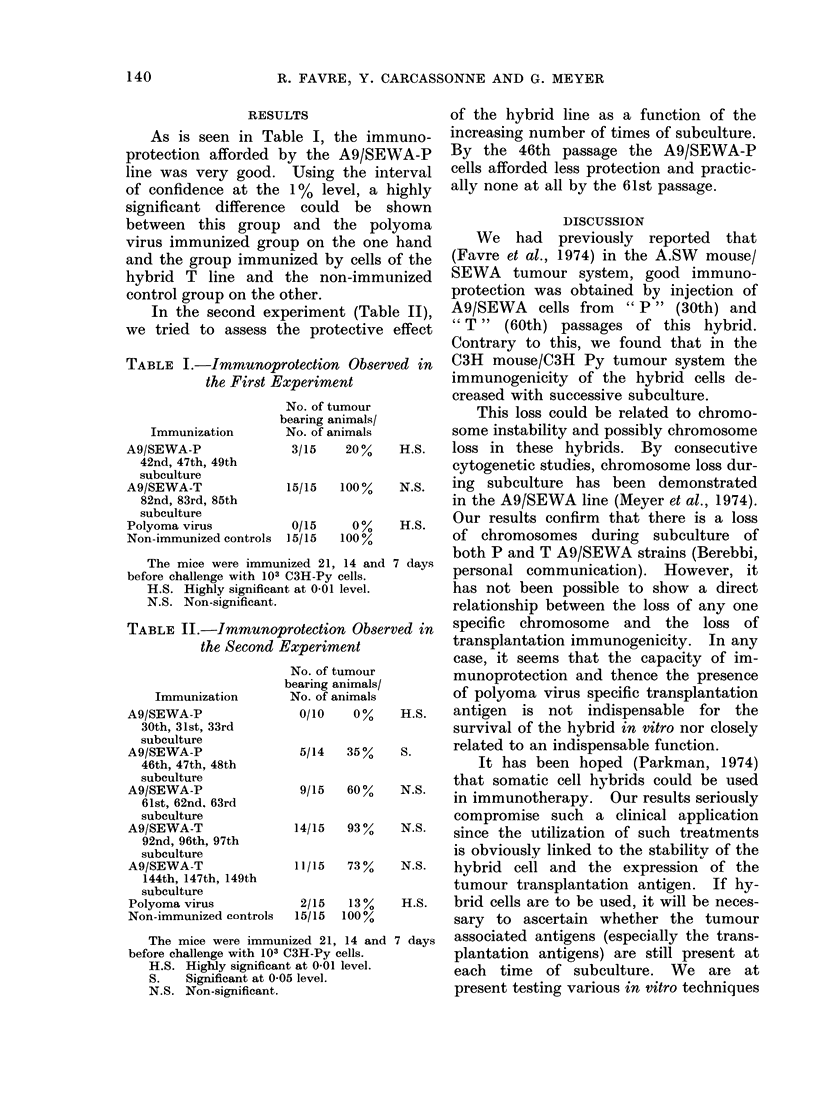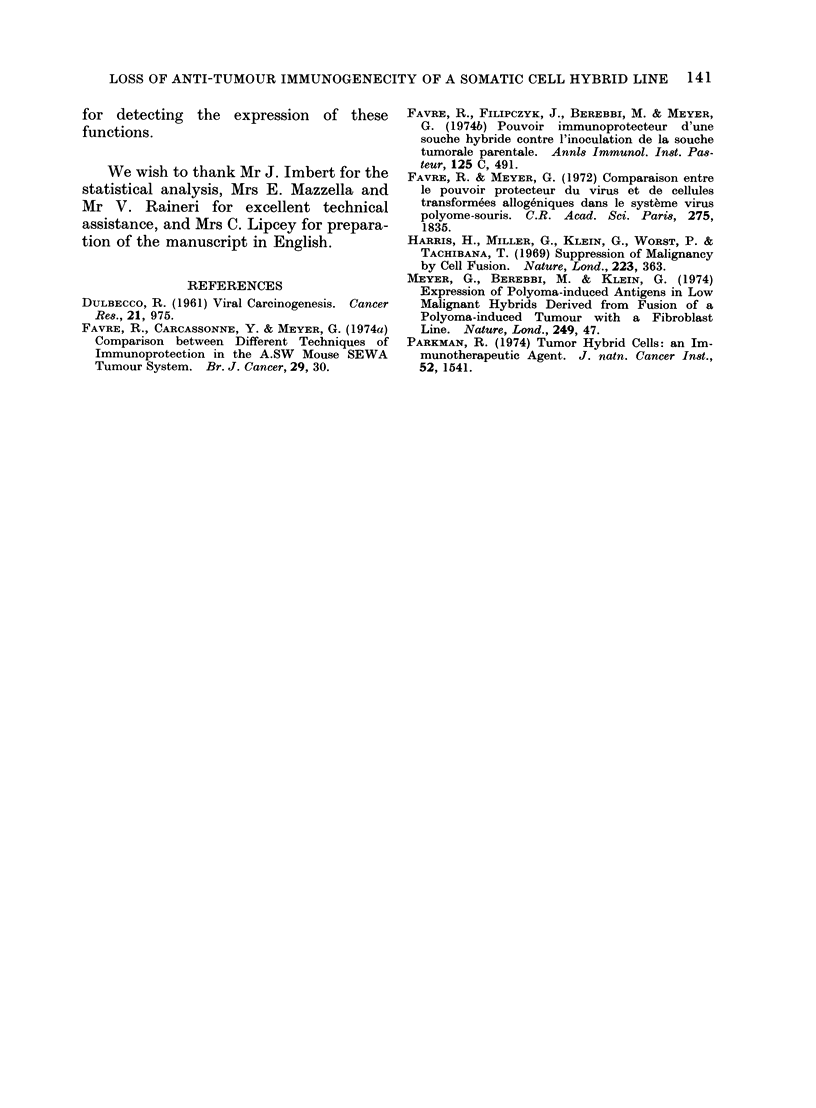# Loss of anti-tumour immunogenicity of a somatic cell hybrid line with increasing subculture.

**DOI:** 10.1038/bjc.1975.140

**Published:** 1975-07

**Authors:** R. Favre, Y. Carcassonne, G. Meyer

## Abstract

Good immunoprotection was afforded by A9/SEWA somatic hybrid cells in the C3H mouse/C3H Py tumour system, confirming results previously obtained in the A.SW mouse/SEWA tumour system. However, in this study the immunogenicity decreased with increasing serial subculture of the hybrid line and concomitant chromosome loss.


*Br. J. Cancer* (1975) **32,** 139

***Short Communication***

### LOSS OF ANTI-TUMOUR IMMUNOGENICITY OF A SOMATIC CELL HYBRID LINE WITH INCREASING SUBCULTURE

R. FAVRE, Y. CARCASSONNE AND G. MEYER

*From the U.*119 *de l'I.N.S.E.R.M.*, 27, *Bd Lei Roure*, 13009 *Marseille*

Received 3 April 1975. Accepted 7 April 1975

**Summary.—Good immunoprotection was afforded by A9/SEWA somatic hybrid cells in the C3H mouse/C3H Py tumour system, confirming results previously obtained in the A.SW mouse/SEWA tumour system. However, in this study the immunogenicity decreased with increasing serial subculture of the hybrid line and concomitant chromosome loss.**

WE HAVE previously shown the interesting occurrence of anti-tumour immunization with somatic hybrid cells (Favre, Carcassonne and Meyer, 1974*a*). In these former experiments, we used the A.SW mouse/polyoma induced SEWA tumour system and protected by immunization with A9/SEWA hybrid cells. However, though we could demonstrate the presence of the tumour antigens characteristic of polyoma virus in these hybrid cells (Favre *et al.*, 1974*b*), we noticed that the transplantation antigen was gradually lost during *in vitro* passaging. This loss does not appear to be fortuitous since two different lines derived from the same hybrid parent displayed the same evolution. Evidently any clinical use of hybrid cells would presuppose the stability of such cells. We therefore describe here further experiments using the complementary H-$2^k$ system (C3H mouse/C3H Py solid tumour), where we tried to assess the protective effect of the A9/SEWA hybrid line as a function of increasing subculture.

### MATERIALS AND METHODS

*Hybrid cells.*—The A9/SEWA hybrid cell line has been fully described elsewhere (Harris *et al.*, 1969). It bears both the H-$2^s$ and H-$2^k$ histocompatibility loci. For this work, 2 series of A9/SEWA cultures, P and T, were used. The difference between these 2 series is that, on reception in our laboratory, the T series was already at a more advanced level of subculture than the P series.

*Tumour cells.*—The C3H-Py line was obtained by *in vitro* transformation of secondary C3H mouse embryo cultures by polyoma virus (Favre and Meyer, 1972). The tumour challenge was effected by means of a dorsal, subcutaneous injection of $10^3$ C3H-Py cells. Morphological and cytogenetic verification that some tumours observed in each group were C3H-Py sarcomata was carried out systematically.

*Polyoma virus.*—The small plaque Toronto strain of polyoma virus grown in secondary mouse embryo cultures was used (Dulbecco, 1961).

*Mice.*—2½ month old inbred C3H mice, bred in our laboratories, were used.

*Immunization schedule.*—Subcutaneous, dorsal injections of $10^6$ A9/SEWA hybrid cells were administered 21, 14 and 7 days before the tumour challenge. A control group received 3 injections of $10^7$ PFU polyoma virus following the same time schedule.

*Karyology.*—The karyotypes were performed as previously described (Meyer, Berebbi and Klein, 1974).

### RESULTS

As is seen in Table I, the immunoprotection afforded by the A9/SEWA-P line was very good. Using the interval of confidence at the 1% level, a highly significant difference could be shown between this group and the polyoma virus immunized group on the one hand and the group immunized by cells of the hybrid T line and the non-immunized control group on the other.

In the second experiment (Table II), we tried to assess the protective effect of the hybrid line as a function of the increasing number of times of subculture. By the 46th passage the A9/SEWA-P cells afforded less protection and practically none at all by the 61st passage.

TABLE I.—*Immunoprotection Observed in the First Experiment*

| Immunization | No. of tumour bearing animals/ No. of animals | | |
|---|---|---|---|
| A9/SEWA-P 42nd, 47th, 49th subculture | 3/15 | 20% | H.S. |
| A9/SEWA-T 82nd, 83rd, 85th subculture | 15/15 | 100% | N.S. |
| Polyoma virus | 0/15 | 0% | H.S. |
| Non-immunized controls | 15/15 | 100% | |

The mice were immunized 21, 14 and 7 days before challenge with $10^3$ C3H-Py cells.

H.S. Highly significant at 0·01 level.

N.S. Non-significant.

TABLE II.—*Immunoprotection Observed in the Second Experiment*

| Immunization | No. of tumour bearing animals/ No. of animals | | |
|---|---|---|---|
| A9/SEWA-P 30th, 31st, 33rd subculture | 0/10 | 0% | H.S. |
| A9/SEWA-P 46th, 47th, 48th subculture | 5/14 | 35% | S. |
| A9/SEWA-P 61st, 62nd, 63rd subculture | 9/15 | 60% | N.S. |
| A9/SEWA-T 92nd, 96th, 97th subculture | 14/15 | 93% | N.S. |
| A9/SEWA-T 144th, 147th, 149th subculture | 11/15 | 73% | N.S. |
| Polyoma virus | 2/15 | 13% | H.S. |
| Non-immunized controls | 15/15 | 100% | |

The mice were immunized 21, 14 and 7 days before challenge with $10^3$ C3H-Py cells.

H.S. Highly significant at 0·01 level.

S. Significant at 0·05 level.

N.S. Non-significant.

### DISCUSSION

We had previously reported that (Favre *et al.*, 1974) in the A.SW mouse/SEWA tumour system, good immunoprotection was obtained by injection of A9/SEWA cells from " P " (30th) and " T " (60th) passages of this hybrid. Contrary to this, we found that in the C3H mouse/C3H Py tumour system the immunogenicity of the hybrid cells decreased with successive subculture.

This loss could be related to chromosome instability and possibly chromosome loss in these hybrids. By consecutive cytogenetic studies, chromosome loss during subculture has been demonstrated in the A9/SEWA line (Meyer *et al.*, 1974). Our results confirm that there is a loss of chromosomes during subculture of both P and T A9/SEWA strains (Berebbi, personal communication). However, it has not been possible to show a direct relationship between the loss of any one specific chromosome and the loss of transplantation immunogenicity. In any case, it seems that the capacity of immunoprotection and thence the presence of polyoma virus specific transplantation antigen is not indispensable for the survival of the hybrid *in vitro* nor closely related to an indispensable function.

It has been hoped (Parkman, 1974) that somatic cell hybrids could be used in immunotherapy. Our results seriously compromise such a clinical application since the utilization of such treatments is obviously linked to the stability of the hybrid cell and the expression of the tumour transplantation antigen. If hybrid cells are to be used, it will be necessary to ascertain whether the tumour associated antigens (especially the transplantation antigens) are still present at each time of subculture. We are at present testing various *in vitro* techniques for detecting the expression of these functions.

We wish to thank Mr J. Imbert for the statistical analysis, Mrs E. Mazzella and Mr V. Raineri for excellent technical assistance, and Mrs C. Lipcey for preparation of the manuscript in English.

### REFERENCES

DULBECCO, R. (1961) Viral Carcinogenesis. *Cancer Res.*, **21**, 975.

FAVRE, R., CARCASSONNE, Y. & MEYER, G. (1974*a*) Comparison between Different Techniques of Immunoprotection in the A.SW Mouse SEWA Tumour System. *Br. J. Cancer*, **29**, 30.

FAVRE, R., FILIPCZYK, J., BEREBBI, M. & MEYER, G. (1974*b*) Pouvoir immunoprotecteur d'une souche hybride contre l'inoculation de la souche tumorale parentale. *Annls Immunol. Inst. Pasteur*, **125** C, 491.

FAVRE, R. & MEYER, G. (1972) Comparaison entre le pouvoir protecteur du virus et de cellules transformées allogéniques dans le système virus polyome-souris. *C.R. Acad. Sci. Paris*, **275**, 1835.

HARRIS, H., MILLER, G., KLEIN, G., WORST, P. & TACHIBANA, T. (1969) Suppression of Malignancy by Cell Fusion. *Nature, Lond.*, **223**, 363.

MEYER, G., BEREBBI, M. & KLEIN, G. (1974) Expression of Polyoma-induced Antigens in Low Malignant Hybrids Derived from Fusion of a Polyoma-induced Tumour with a Fibroblast Line. *Nature, Lond.*, **249**, 47.

PARKMAN, R. (1974) Tumor Hybrid Cells: an Immunotherapeutic Agent. *J. natn. Cancer Inst.*, **52**, 1541.